# Study of gas production from shale reservoirs with multi-stage hydraulic fracturing horizontal well considering multiple transport mechanisms

**DOI:** 10.1371/journal.pone.0188480

**Published:** 2018-01-10

**Authors:** Chaohua Guo, Mingzhen Wei, Hong Liu

**Affiliations:** 1 Key Laboratory of Tectonics and Petroleum Resources (China University of Geosciences), Ministry of Education, Wuhan, China; 2 Department of Geological and Petroleum Engineering, Missouri University of Science and Technology, Rolla, MO, United States of America; 3 Department of Petroleum and Natural Gas Engineering, Chongqing University of Science and Technology, Chongqing, China; College of Agricultural Sciences, UNITED STATES

## Abstract

Development of unconventional shale gas reservoirs (SGRs) has been boosted by the advancements in two key technologies: horizontal drilling and multi-stage hydraulic fracturing. A large number of multi-stage fractured horizontal wells (MsFHW) have been drilled to enhance reservoir production performance. Gas flow in SGRs is a multi-mechanism process, including: desorption, diffusion, and non-Darcy flow. The productivity of the SGRs with MsFHW is influenced by both reservoir conditions and hydraulic fracture properties. However, rare simulation work has been conducted for multi-stage hydraulic fractured SGRs. Most of them use well testing methods, which have too many unrealistic simplifications and assumptions. Also, no systematical work has been conducted considering all reasonable transport mechanisms. And there are very few works on sensitivity studies of uncertain parameters using real parameter ranges. Hence, a detailed and systematic study of reservoir simulation with MsFHW is still necessary. In this paper, a dual porosity model was constructed to estimate the effect of parameters on shale gas production with MsFHW. The simulation model was verified with the available field data from the Barnett Shale. The following mechanisms have been considered in this model: viscous flow, slip flow, Knudsen diffusion, and gas desorption. Langmuir isotherm was used to simulate the gas desorption process. Sensitivity analysis on SGRs’ production performance with MsFHW has been conducted. Parameters influencing shale gas production were classified into two categories: reservoir parameters including matrix permeability, matrix porosity; and hydraulic fracture parameters including hydraulic fracture spacing, and fracture half-length. Typical ranges of matrix parameters have been reviewed. Sensitivity analysis have been conducted to analyze the effect of the above factors on the production performance of SGRs. Through comparison, it can be found that hydraulic fracture parameters are more sensitive compared with reservoir parameters. And reservoirs parameters mainly affect the later production period. However, the hydraulic fracture parameters have a significant effect on gas production from the early period. The results of this study can be used to improve the efficiency of history matching process. Also, it can contribute to the design and optimization of hydraulic fracture treatment design in unconventional SGRs.

## Introduction

Shale gas reservoirs (SGRs) are organic-rich formations, which serve as both reservoir and source rock together. Gas is stored as free gas in natural fractures and matrix pore structure, and as adsorbed gas on the surface of shale matrix and in organic materials [1, 2]. Exploitation of unconventional SGRs has become an important component to secure natural gas supply of North America. Due to the improved stimulation techniques and multi-stage horizontal well drilling, economic development of ultra-low permeability SGRs becomes viable [3]. As shale formations have extremely low permeability which is about 54 to 150 nano-Darcy [3–5], economical development of shale had been regarded as impossible for a very long time until hydraulic fracturing technic has been used. Then, shale gas gradually became an important part of natural gas production and experienced a rapid growth since 2005. The combination of horizontal well and multi-stage hydraulic fracturing treatment can significantly improve production rate by enhancing the reservoir permeability and creating stimulated reservoir volume.

Accurate modeling of hydraulic fracturing treatment in SGRs is complicated due to the complex nature of hydraulic fracture growth, poor understanding of the treatment process, and lack of good quality reservoir information [1, 2, 6]. Therefore, reservoir simulation is the preferred method to predict and evaluate well performance of SGRs. Compared with conventional reservoirs, SGRs contain a large portion of nano pores and micro fractures. The key problem in simulating fractured SGRs is how to handle the fracture-matrix interaction. Yu and Sepehrnoori (2014) have used CMG software to optimize shale gas production [6, 7]. They proposed an efficient reservoir-simulation approach by integrating the commercial simulator, economic model, design of experiment, and response-surface methodology with a global optimization search engine. Jahandideh and Jafarpour (2016) have constructed a single porosity model to study the effect of heterogeneity in shale fracability on the design and optimization of the hydraulic fracturing process [8]. Many different models have been proposed to simulate fluid flow in fractured reservoirs [9–14]. These methods include: (1) equivalent continuum model [8, 15]; (2) dual porosity model, including dual porosity single permeability model, and dual porosity dual permeability model [16, 17]; (3) Multiple Interaction Continua (MINC) method [18, 19]; (4) Multiple porosity model [20–22]; and (5) Discrete fracture model [23–24]. Though the discrete fracture model is more rigorous than the first three models, it is still limited in applications to field study due to computational intensity and lack of detailed knowledge of fracture distribution [20].

*Equivalent continuum model*. In the equivalent continuum model, the fractured porous media is considered as a continuum media. The properties of the fracture arrays, such as direction, location, permeability, porosity, etc., are averaged into the whole porous media. This method has not been widely used in SGR simulation as it is not easy to obtain the averaged permeability tensor and it is too conceptual. Moridis et al. (2010) have constructed effective continuum SGR simulation model with considering muti-component gas adsorption [25]. In the effective continuum model, the SGR is discretized and the fractures are characterized with grid cells as single plannar planes or network of planar planes [26, 27].

*Dual Porosity Model*. Dual porosity model is widely used to simulate the fluid flow in fractured porous media and many scholars have published a lot of literature based on dual porosity model [12, 16, 17, 28, 29]. There are two interacting media: the matrix blocks with high porosity and low permeability, and the fracture network with low porosity and high conductivity. According to fluid flow situation in the matrix, the dual porosity model can be classified into dual porosity single permeability (DPSP) model and the dual porosity dual permeability (DPDP) model. In the DPSP model, the flow only occur through the fracture system and matrix are treated as the spatially distributed sinks or sources to the fracture system [30]. Watson et al. (1990) have performed production data analysis for Devonian SGR using DPM and presented analytical reservoir production models for history matching and production forecasting [31]. Bustin et al. (2008) have constructed a 2D dual porosity model to simulate SGRs which uses both experimental and field data as model input parameters [10]. Ozkan et al. (2010) have developed a dual-mechanism dual-porosity model to simulate the linear flow for fractured horizontal well in SGRs [12]. In the dual porosity dual permeability model, the flow in the matrix is considered additionally [22]. Moridis et al. (2010) constructed a dual permeability model and compared it with dual porosity model and effective continuum model [25]. Results showed that dual permeability model offered the best production performance.

*Multiple Interaction Continua method*. As an extension for dual porosity model, Pruess (1985) have proposed a multiple interaction continua method (MINC) for more accurately characterizing the interaction between matrix and fracture [18]. The matrix system in every grid block for MINC method will be subdivided into a sequence of nested rings which can more accurately calculate the interblock fluid flow. Compared with the dual porosity model, the MINC method is able to describe the gradient of pressures, temperatures, or concentrations near matrix surface and inside the matrix, which can provide better numerical approximation for the transient fracture matrix interaction [30]. Wu et al. (2009) have proposed the generalized mathematical model to simulate multiphase flow in tight fractured reservoirs using MINC method with considering following mechanisms: Klinkenberg effect, non-Newtonian behavior, non-Darcy flow, and rock deformation [30].

*Multiple Porosity Model*. Some scholars also proposed multiple porosity model in which matrix is further separated into two or three parts with different properties. Alahmadi (2010) has constructed triple porosity model considering two different fractures in the formation and derived the Laplace solution for the linear flow [32]. Civan et al. (2011) proposed the qual-porosity model to simulate SGRs, including organic matter, inorganic matter, natural, and hydraulic fractures [20]. Dehghanpour et al. (2011) have proposed the triple porosity model to simulate SGRs by incorporating micro fractures into the dual porosity models [21]. They assumed that the matrix blocks in the dual porosity model is composed of sub-matrix with nano Darcy permeability, and micro fractures with milli to micro Darcy permeability. Yan et al. (2013) presented a micro-scale model [22]. In Yan’s model, the shale matrix was classified into inorganic matrix and organic matrix. The organic part was further divided into two parts basing on pore size of kerogen: organic materials with vugs and organic materials with nanopores. Therefore, there are four different continua in this model: nano organic matrix, vug organic matrix, inorganic matrix, and micro fractures. Ghamdi and Ershaghi (1996) have proposed linear triple porosity model and derived the solution in Laplace space. However, the model did not consider gas desorption and diffusion [33].

*Discrete Fracture Model*. In the discrete fracture model, the fractures are discretely modeled. Each fracture is characterized as a geometrically well-defined entity. When modeling SGRs using discrete fracture model, gas flow from the matrix to the discrete fractures by diffusion and desorption [13, 34, 35]. Gong et al. (2011) have proposed a systematic methodology of constructing a discrete fracture model based on microseismic interpretations for SGRs with considering multiple mechanisms, such as gas adsorption, non-Darcy effect, etc [34]. Mayerhoffer et al. (2006) have investigated the fluid flow in fractured SGRs considering stimulated reservoir volume (SRV) using explicit fracture network [35]. Cipolla et al. (2009) have investigated the effect of hydraulic fracturing parameters on shale gas production [27, 36], including fracture conductivity, fracture spacing, and gas desorption. However, due to the scale of fracture aperture (mm scale), horizontal well (cm scale), and reservoir (Km scale), the computational cost is extremely huge due to the multi-scale physics coupling.

The discrete fracture model is more rigorous. However, application of this method to reservoir scale is limited due to computational intensity and lack of detailed knowledge of fracture matrix properties and spatial distribution in reservoirs [20, 30]. The dual porosity model can deal with the fracture matrix interaction more easily and is less computationally demanding than discrete fracture method. Thus, the dual porosity model is the main approach for modeling fluid and heat flow in fractured porous media [30].

Many scholars have conducted numerical simulation of SGRs using above mentioned methods. However, rare simulation work have been conducted for multi-stage hydraulic fractured SGRs. Most of them use well testing methods which have too many simplifications. Also, no systematical work has been conducted considering all reasonable transport mechanisms. And there are very few work on sensitivity studies of uncertain parameters using real parameter range. Hence, a detailed and systematic study of reservoir simulation with MSFHW is still necessary. In this paper, the numerical simulation model for SGRs with multi-stage hydraulic fracturing horizontal well was constructed based on the dual porosity model. Mass balance equations for both matrix and fracture systems were constructed using the dusty gas model. In the matrix, Knudsen diffusion, gas desorption, and viscous flow were considered. Gas desorption was characterized by the Langmuir isothermal equation. In the fracture, viscous flow and non-Darcy slip permeability were considered. The model got solved using finite element software COMSOL. The comprehensive sensitivity analysis was conducted, and detailed investigation were done regarding their impact on the SGRs production performance. The rest of this paper are organized as follows: Section 2 has presented the gas flow mechanisms in SGRs. In section 3, the apparent permeability for gas flow in shale matrix and fracture system have been derived. In section 4, the mathematical model for gas production from shale reservoirs with multi-stage hydraulic fracturing horizontal well has been constructed. Section 5 provided the model verification using the production data from Barnett shale. And lastly, sensitivity analysis has been conducted for hydraulic fracture parameters based on the synthetic model.

## Fluid flow mechanisms in tight shale reservoirs

Due to the complexity of flow in SGRs, three flow mechanisms should be considered in the dual-permeability model: viscous flow, Knudsen diffusion, slip flow, and gas adsorption.

### Viscous flow

This is the region where Knudsen number (The ratio of gas mean free path to pore throat diameter) has fairly low values such as in the conventional reservoirs with pore diameter in micrometers. In this Knudsen number range, the free path of the gas is smaller than the pore diameter (Knudsen number is far less than 1), so the motion of the gas molecules is mainly affected by the collision between molecules. The molecule’s collision with the pore walls are negligible [37]. The gas flow is driven by the pressure gradient between the single component gas, which can be characterized using the Darcy law:

$$J_{v}=-\frac{{\rho_{m}k}_{mi}}{\mu_{g}}(\nabla p_{m})$$ (1)

where *J*_*v*_ is the mass flow (*kg*/*m*^2^⋅*s*)), *ρ*_*m*_ is gas density in bedrock (*kg*/*m*^3^), *k*_*mi*_ is the intrinsic permeability of bedrock (m^2^), *μ*_*g*_ is the gas viscosity (Pa⋅s), *p*_*m*_ is pore pressure in the bedrock (Pa).

### Knudsen diffusion

This is the region where the Knudsen number is larger than 1 where the pore diameter is small enough so that the mean free path of the gas is close to the pore diameter. Under this Knudsen range, the gas flow is dominated by the collision between the gas molecules and the wall surface. The gas mass flow can be characterized using the Knudsen diffusion equation [37], which is shown in Eq (2):

$$J_{k}=-M_{g}D_{km}(\nabla C_{m})$$ (2)

where J_k_ is the mass flow caused by Knudsen diffusion (kg/(m^2^·s)), *M*_*g*_ is the gas molar mass (kg/mol), *D*_*km*_ is the diffusion coefficient of the bedrock (m^2^/s), *C*_*m*_ is the gas mole concentration (mol/ m^3^).

### Slip flow

In low-permeability formations (such as SGRs) or when the pressure is very low, gas slip flow (or Klinkenberg effect) cannot be ignored when studying gas transport in tight reservoirs [38–40]. Under such kind of flow conditions, gas permeability depends on gas pressure, which can be expressed as follows:

$$\vec{k_{a}}=\vec{k_{i}}(1+\frac{b_{k}}{p})$$ (3)

where *k*_*a*_ is the apparent permeability, $\vec{k_{i}}$ is the intrinsic permeability, *b* is the slip coefficient. Many scholars have proposed different expressions for the slip coefficient which are listed in the Table 1 [5].

**Table 1:** Knudsen’s permeability correction factor for tight porous media [5].

Model	Correlation factor
Klinkenberg	$b_{k}=4c\lambda \bar{P}/r$
Heid et al.	*b*_*k*_ = 11.419(*k*_∞_)^−0.39^
Jones and Owens	*b*_*k*_ = 12.639(*k*_∞_)^−0.33^
Sampath and Keighin	*b*_*k*_ = 13.851(*k*_∞_/*ϕ*)^−0.53^
Florence et al.	*b*_*k*_ = *β*(*k*_∞_/*ϕ*)^−0.5^
Civan	*b*_*k*_ = 0.0094(*k*_∞_/*ϕ*)^−0.5^

### Gas adsorption and desorption

Compared with conventional gas reservoirs, SGRs can produce a large amount of gas from desorption during the depressurization process [1, 2]. Gas desorption is an important mechanism and factor affecting ultimate shale gas recovery. Neglecting the gas desorption effect will underestimate gas potential, especially in shale formations with higher Total Organic Content [41]. Gas desorption occurs when a pressure difference exists in organic grids (Kerogen). With free gas production, the pressure in the pores will decrease, which results in the pressure difference between the bulk matrix and the pores. Due to this pressure drop, gas will desorb from the surface of bulk matrix. The most commonly used empirical model which characterizes sorption and desorption of gas in shale and provides a reasonable fit to most experimental data is the Langmuir single-layer isotherm model [42, 43]. Based on the Langmuir equation (Eq (4)), the amount of gas adsorbed on the rock surface can be calculated.

$$V_{ads}=\frac{V_{L}\cdot p}{p_{L}+p}$$ (4)

where *V*_*L*_ is the gas Langmuir volume denoting the amount of gas adsorbed at infinite pressure *p*_∞_, scf/ton; *P*_*L*_ is the gas Langmuir pressure corresponding to the pressure at which half of the Langmuir volume v_L_ is reached, psi; *P*_*g*_ is the in-situ gas pressure in the pore system, psi. Langmuir volume means the maximum amount of gas that can be adsorbed on the rock surface under infinite pressure. Langmuir pressure is the pressure when the amount of gas adsorbed is half of the Langmuir volume. These two parameters play important roles in the gas desorption process. For different SGRs, the differences of Langmuir volume and Langmuir pressure can lead to distinct trend of gas content.

The adsorption gas volume per unit bulk volume can be expressed in Eq (5):

$$q_{a}=\frac{\rho_{s}M_{g}}{V_{std}}q_{std}=\frac{\rho_{s}M_{g}}{V_{std}}\frac{V_{L}p_{m}}{p_{L}+p_{m}}$$ (5)

where *q*_*a*_ is the adsorption gas per unit area of shale surface, kg/m^3^; *V*_*std*_ is the mole volume under standard condition (0°C, 1atm), m^3^/mol; *q*_*std*_ is the adsorption volume per unit mass of shale, m^3^/kg; *V*_*L*_ is the Langmuir volume, m^3^/kg; *p*_*L*_ is the Langmuir pressure, *P*_*a*_; *ρ*_*s*_ is the density of shale core, kg/m^3^.

## Apparent gas permeability in the matrix and fracture system

### Apparent gas permeability in the shale matrix

Some empirical models have been developed to account for slip-flow and Knudsen diffusion in the form of apparent permeability, including correlations developed from the Darcy matrix permeability [12]; correlations developed based on flow mechanisms [44, 45]; correlations as a function of Knudsen number based on Beskok and Karniadakis’s work [40, 46]; and semi-empirical analytical models by Moridis et al. (2010) [25]. This research adopted Javadpour’s method which considered slip flow, viscous flow and Knudsen diffusion [44]. The apparent permeability is given as follows [4]:

$$\vec{k_{m}}=\vec{k_{mi}}\left(1+\frac{b_{m}}{p_{m}}\right)$$ (6)

$$b_{m}=\frac{16\mu }{3000r}{\left(\frac{8\pi RT}{M}\right)}^{0.5}+{\left(\frac{8\pi RT}{M}\right)}^{0.5}\frac{\mu }{r}(\frac{2}{\alpha }-1)$$ (7)

where *α* is the tangential momentum accommodation coefficient which characterizes the slip effect. *α* is a function of wall surface smoothness, gas type, temperature and pressure which varies in a range from 0 to 1. For simplification, in this paper, it is set as 0.8.

### Apparent gas permeability in the shale fractures

In this study, Knudsen diffusion and viscous flow are considered for gas flow in the fracture system. The mass flux can be expressed as the summation of the two mechanisms. In our previous work Guo et al. (2015) [4], we already derived the fracture apparent permeability, which is shown as follows:

$$\vec{k_{f}}=\vec{k_{fi}}\left(1+\frac{b_{f}}{p_{f}}\right)$$ (8)

where

$$b_{f}=\frac{D_{kf}\mu_{g}}{k_{fi}}$$ (9)

$$D_{kf}=\frac{4k_{fi}}{2.8284\sqrt{\frac{k_{fi}}{\phi_{f}}}}\sqrt{\frac{\pi RT}{2M_{g}}}$$ (10)

where *p*_*f*_ is the fracture pressure, *k*_*fi*_ is the initial fracture permeability, *k*_*f*_ is the apparent fracture permeability, *b*_*f*_ is the Klinkenberg coefficient for the fracture system, *D*_*kf*_ is the Knudsen diffusion coefficient for the fracture system, ∅_f_ is the fracture porosity.

## Numerical simulation model for shale gas reservoirs with MsFHW

### Model assumptions

Assumptions are as follows:

1. The flow is isothermal;
2. There are only one component, one phase flow in the shale reservoir;
3. The gas diffuse quickly and can achieve phase equilibrium instantaneously;
4. Formation rocks are incompressible and the porosity change due to rock deformation is ignored;
5. The gas in the shale reservoir can be assumed ideal gas with gas deviation factor equal to 1;
6. Gas adsorption-desorption kinetics obey Langmuir isotherm equation.

### Mathematical model

(1) Continuity equation

**Fig 1:**
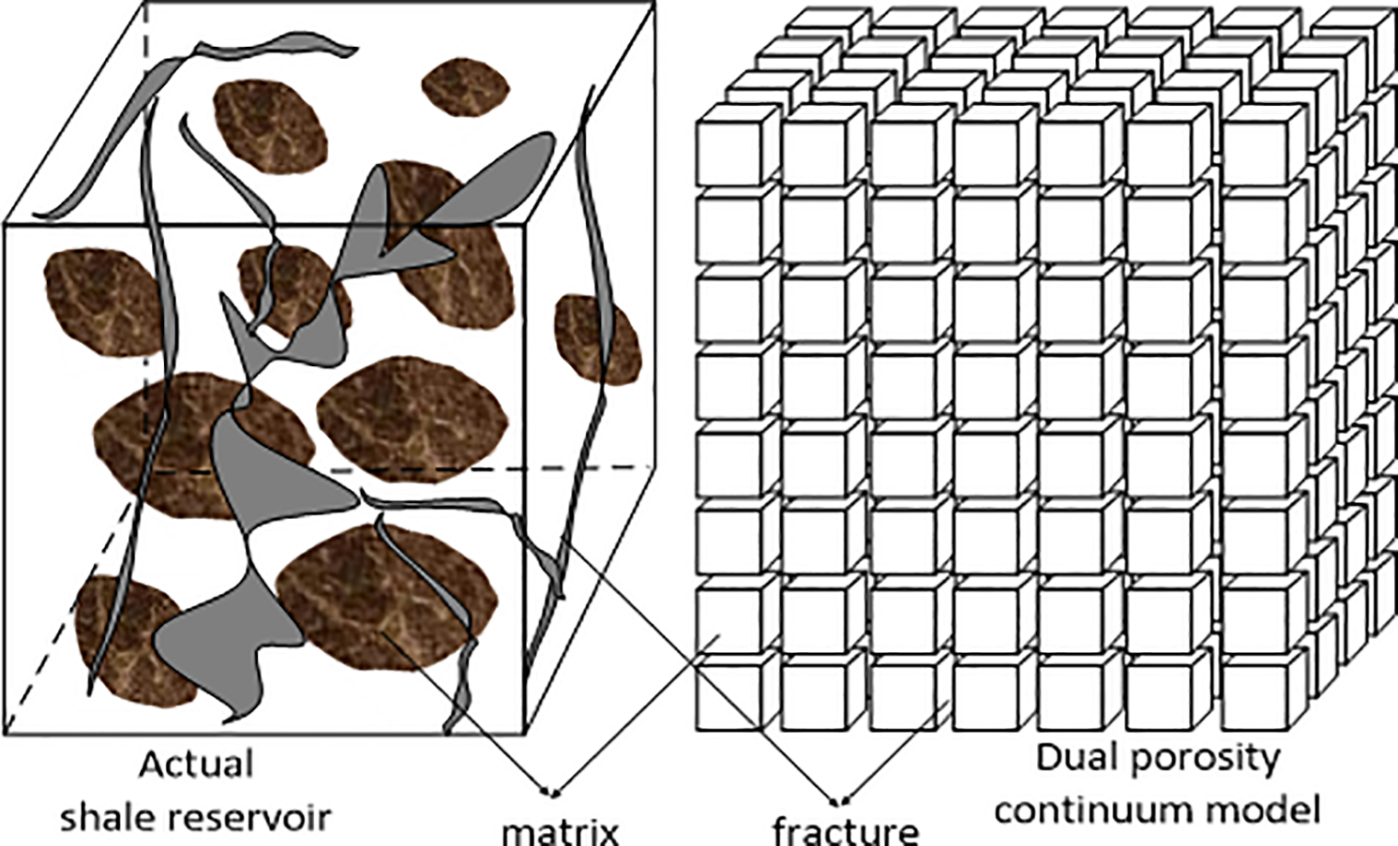
Diagram of dual porosity model.

In the dual porosity model, there exist two mass balance equations corresponding for fracture and matrix systems respectively as indicated by Warren and Root (1963) [17]. The diagram of dual porosity model is shown in the Fig 1.

The continuity equations for the matrix and fracture system are as follows:

In the matrix:

$${\left(\frac{dM}{dt}\right)}_{m}+{\left(\nabla \cdot (\rho \vec{v_{m}})\right)}_{m}=Q_{m}$$ (11)

In the fracture:

$${\left(\frac{dM}{dt}\right)}_{f}+{\left(\nabla \cdot (\rho \vec{v_{f}})\right)}_{f}=Q_{f}$$ (12)

The first term on the left side, *M*, is the mass accumulation term; the second term on the left side, $\vec{v}$, is the flow vector term; and the right side, *Q*, is the source/sink term.

In the matrix, there are adsorption gas and free gas co-existed. So, in the matrix, the mass accumulation term is the sum of adsorption gas and free gas. The mass accumulation term is shown as follows. The detailed derivation can be found in Guo et al. (2015) [4].

$${\left(\frac{dM}{dt}\right)}_{m}=\left[\frac{M}{ZRT}\varphi_{m}+\frac{(1-\varphi_{m})M_{g}p_{L}V_{L}\rho_{s}}{V_{std}{\left(p_{L}+p_{m}\right)}^{2}}\right]\frac{\partial p_{m}}{\partial t}$$ (13)

In the fracture system, only free gas existed, so the mass accumulation term can be described as follows:

$${\left(\frac{dM}{dt}\right)}_{f}=[\frac{M}{ZRT}\varphi_{f}]\frac{\partial p_{f}}{\partial t}$$ (14)

The meanings of each symbol are listed in the Nomenclature section.

(2) Motion equation

In the matrix and fractures, the permeability can be characterized using the modified Darcy’s model. The motion equations are as follows:

$$\vec{v_{m}}=-\frac{K_{mg}}{\mu_{g}}[\nabla (p_{mg}-\rho_{g}gD)]$$ (15)

$$\vec{v_{f}}=-\frac{K_{fg}}{\mu_{g}}[\nabla (p_{fg}-\rho_{g}gD)]$$ (16)

In the matrix, *K*_*mg*_ = *k*_*mi*_(1 + *b*_*m*_/*p*_*m*_), where *b*_*m*_ is shown in Eq (7).

In the fracture, *K*_*fg*_ = *k*_*mi*_(1 + *b*_*f*_/*p*_*f*_), where *b*_*f*_ is shown in Eq (9).

(3) Gas production from Multi-stage hydraulic fracturing horizontal well

In the horizontal well with multi-stage transverse hydraulic fractures, the fractures are the main flow channels for the fluid flow from the shale micro-fractures into the horizontal wellbore. Fundamentally different from the gas production from SGRs with a vertical well [4], SGRs with multi-stage horizontal well are more complicated. The gas production of a horizontal wellbore is the sum of the gas production from each hydraulic fracture. For gas production from one single fracture, we assumed each fracture as a horizontal well along the y direction. This is the difference between the vertical well and multi-stage horizontal well. Peaceman’s model was used in this study [47]. The following equation assumes the horizontal well is along the x direction:

$$q_{p}=\frac{k_{f}\rho_{f}}{\mu_{g}}\frac{2\pi d_{x}}{\ln\left(r_{e}/r_{w}\right)+s}(\bar{p_{f}}-p_{wf})$$ (17)

where *p*_*wf*_ is the bottom-hole pressure, *d*_*x*_ is the fracture length along the x direction, and *r*_*e*_ is the effective radius and can be expressed as:

$$r_{e}=\left\{ \begin{array}{c} 0.208\Delta y\;when\;\Delta y=\Delta z,K_{y}=K_{z}\\ 0.28\frac{{\left[{\left(K_{y}/K_{z}\right)}^{1/2}\Delta z^{2}+{\left(K_{z}/K_{y}\right)}^{1/2}\Delta y^{2}\right]}^{0.5}}{{\left(K_{y}/K_{z}\right)}^{1/4}+{\left(K_{z}/K_{y}\right)}^{1/4}}\;other \end{array}\right.$$ (18)

(4) Numerical simulation model

By substituting the motion equations into continuity equation, the complete numerical simulation model was derived which is composed of flow equation, boundary conditions and initial condition.

Equation in the matrix system:

$$[\frac{M}{ZRT}\phi_{m}+\frac{(1-\phi_{m})M_{g}p_{L}V_{L}\rho_{s}}{V_{std}{\left(p_{L}+p_{m}\right)}^{2}}]\frac{\partial p_{m}}{\partial t}-\nabla \cdot [\frac{M}{ZRT}[\frac{k_{mi}(p_{m}+b_{m})}{\mu_{g}}(\nabla p_{m})]]=-T\;$$ (19)

Equation in the fracture system:

$$[\frac{M}{ZRT}\phi_{f}]\frac{\partial p_{f}}{\partial t}-\nabla \cdot \left[\frac{M}{ZRT}[\frac{p_{f}k_{f}(p_{f}+b_{f})}{\mu_{g}}(\nabla p_{f})]\right]=T-Q_{p}$$ (20)

Where T is the gas transfer between the matrix and fracture systems [16, 17] is represented in the Eq (24):

$$T=\frac{\left(k_{m}\rho_{g}\sigma (p_{m}-p_{f}\right)}{\mu }$$ (21)

where $\sigma =4(\frac{1}{L_{x}^{2}}+\frac{1}{L_{y}^{2}}+\frac{1}{L_{z}^{2}})$ is the crossflow coefficient between the matrix and fracture systems, and *L*_*x*_, *L*_*y*_, *L*_*z*_ are the fracture spacing in the *x*, *y*, *z* directions. In the fracture system, there exists a source term which flows into fracture from the matrix and a sink term which flows out of fracture into the wellbore. *Q*_*p*_ is the sum of gas production from the fractures of the multi-stage hydraulic fractured horizontal well.

Initial condition: *p*_*m*_|_*t* = 0_ = *p*_*f*_|_*t* = 0_ = *p*_*i*_

Boundary condition for matrix: ${F_{m}\cdot n|}_{\Gamma_{1}}$ = 0 (${\frac{\partial p}{\partial n}|}_{\Gamma_{1}}=0$)

Boundary condition for fracture: ${F_{f}\cdot n|}_{\Gamma_{1}}=0\;({\frac{\partial p}{\partial n}|}_{\Gamma_{1}}=0)$, ${p_{f}(x,y,t)|}_{\Gamma_{2}}=p_{wf}$

## Model verification

**Fig 2:**
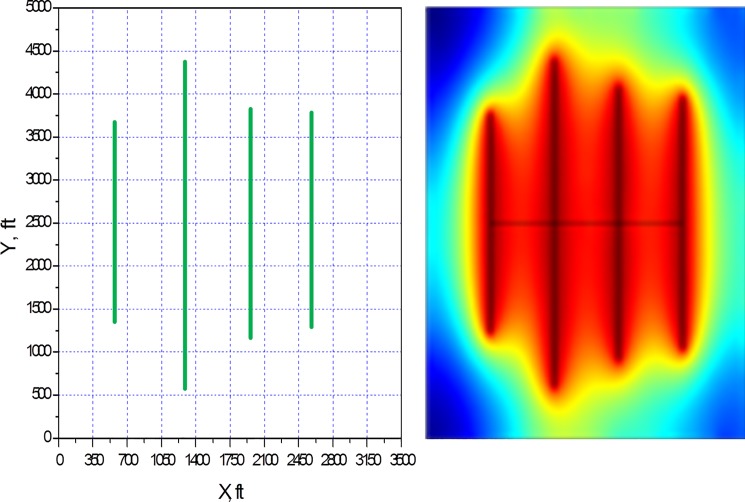
(a) Four stage hydraulic fracturing horizontal well in Barnett shale. (b) Simulation model for four stage hydraulic fracturing horizontal well.

**Fig 3:**
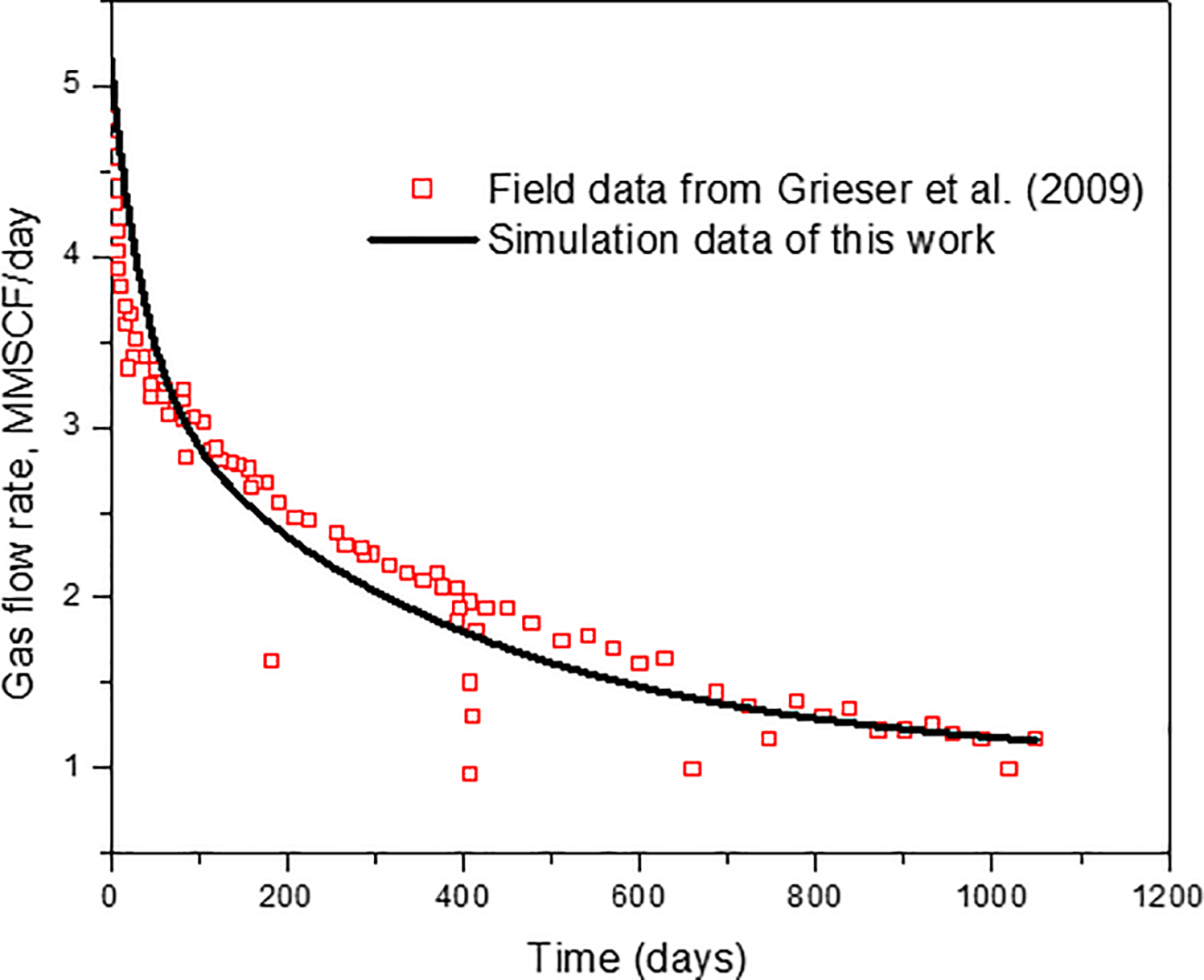
Comparison between simulation results and the field data of Barnett shale.

Once the shale gas reservoir numerical simulation model including M_S_FHW is constructed, it requires validation with field production data to ensure the reliability of simulation results. After validation, it can be used to perform sensitivity studies and production forecasting for a long-time period. To verify the accuracy of the simulation model, comparisons are made with the actual field production data from Barnet shale [1, 2, 48]. Using these data, Grieser et al. (2009) have developed a 3-D multi-stage hydraulic fracturing horizontal well simulator for north Texas Barnett shale gas reservoir [48]. However, Knudsen diffusion and nano scale gas transport permeability correlation has not been considered in that work. The history match was achieved by adjusting matrix and fracture permeability. Yu and Sepehrnoori (2014) have performed history matching for field gas production data from the Barnett Shale and Marcellus Shale using CMG software [1]. The effect of gas desorption on gas recovery has been studied. However, due to limitations of the CMG software, Knudsen diffusion and nano pore confinement cannot be considered in the model. Considering the previous history match work, in this work, the research team will directly input the parameters from their history match results [1] into our mathematical model to check the reliability of our model. A single phase dual-permeability SGR simulation model was constructed in this paper by considering multiple flow mechanisms: Knudsen diffusion, slip flow, viscous flow, and gas adsorption/desorption. The Langmuir isotherm was used to simulate the gas desorption process. The mathematical model has been solved using Comsol Multiphysics, which is a commercial software based on the finite element method. For more strictly evaluating the validity and general applicability of our simulation model, we directly used the parameters from Yu et al., (2014) [1]. The model dimension is 3500ft in length, 5000ft in width and 400ft in height. In the middle of the reservoir, a horizontal well with 4 stages of hydraulic fractures was constructed as shown in Fig 2. The reservoir and hydraulic fracturing parameters are listed in the Table 2. The comparison results between the simulation results and the field data are shown in Fig 3. As can be seen from Fig 3, simulation results provide a good match with the field data. Compared with the simulation results from CMG software in Yu et al. (2014), the simulation results are not completely identical to the simulation results from the CMG software. This is due to differences in the gas transport mechanisms and calculation methods between the models. However, it can be seen that the simulation results in this work yield a reasonable match with the actual field production data from the Barnett shale.

**Table 2:** Simulation parameters for Barnett shale case [1].

Parameters	Barnett Shale case	Unit
Model dimensions (LxWxH)	3500x5000x400 (1066.8x1524x121.9)	ft (m)
Initial reservoir pressure	3800 (2.62x10^7^)	psi (Pa)
Bottom-hole pressure	1500 (1.03x10^7^)	psi (Pa)
Production period	3 (9.46x10^7^)	year (s)
Reservoir temperature	180 (82)	^o^F (^o^C)
Gas viscosity	0.02 (0.00002)	cP (Pa*s)
Bulk density	158 (2530.92)	lb/ft^3^ (kg/m^3^)
Reservoir top depth	7000 (2133.6)	ft (m)
Langmuir pressure	650 (4.48 x10^6^)	psi (Pa)
Langmuir volume	96 (0.0027)	SCF/ton (m^3^/kg)
Fracture conductivity	9 (2.7x10^-15^)	md-ft (m^2^-m)
Fracture height	400 (121.9)	ft (m)
Matrix permeability	0.00035 (3.5x10^-19^)	md (m^2^)
Matrix porosity	0.04	fraction
Horizontal wellbore length	2052 (625.4)	ft (m)

## Sensitivity analysis

**Fig 4:**
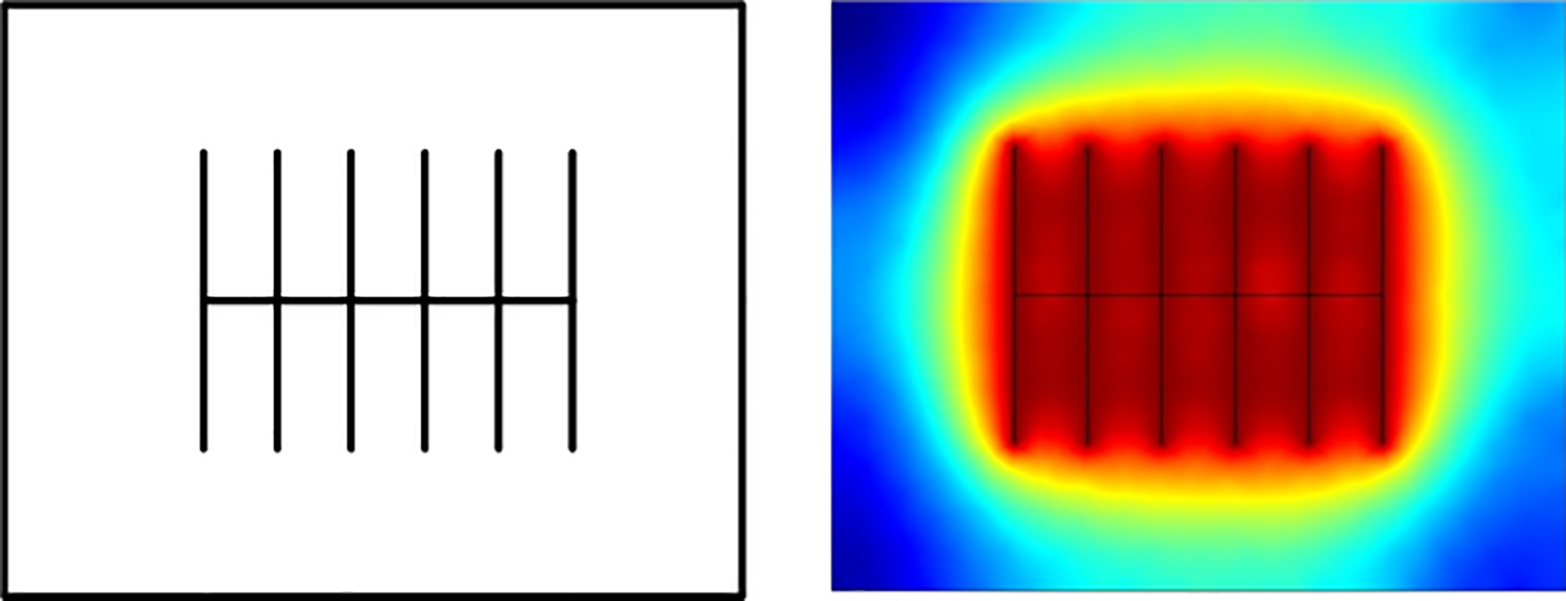
Synthetic model for production performance study.

In order to conduct sensitivity analysis, we constructed another SGR simulation model with a volume of 2500 ft x 2000 ft x 300 ft, which is called the synthetic model. The parameters used in the synthetic model have been listed in the Table 3. In the middle of the reservoir, there is a horizontal well with six stages of hydraulic fractures which is shown in Fig 4. Different from our previous work [4], this work focuses on the effect of reservoir and hydraulic fracture parameters. The effect of gas flow mechanisms on the gas production performance has already been illustrated in our previous work. In this work, sensitivity analysis was constructed based on the synthetic model and two categories of parameters’ effect on the production performance of SGRs have been studied: the reservoir parameters and the hydraulic fracture parameters. For the reservoir parameters, we have studied the effect of matrix permeability and matrix porosity on the production performance of the SGR. For the hydraulic fracture parameters, two parameters have also been studied: fracture spacing and fracture half-length.

**Table 3:** Parameters used in the synthetic model for production performance study.

Parameters	Barnett Shale case	Unit
Model dimensions (LxWxH)	2500x2000x300 (762x609.6x91.44)	ft (m)
Initial reservoir pressure	2400 (1.65x10^7^)	psi (Pa)
Bottom-hole pressure	500 (3.45x10^6^)	psi (Pa)
Production period	20 (6.31x10^8^)	year (s)
Reservoir temperature	200 (93.3)	^o^F (^o^C)
Gas viscosity	0.02 (0.00002)	cP (Pa*s)
Bulk density	158 (2530.92)	lb/ft^3^ (kg/m^3^)
Reservoir top depth	6800 (2072.6)	ft (m)
Langmuir pressure	650 (4.48 x10^6^)	psi (Pa)
Langmuir volume	100 (0.0028)	SCF/ton (m^3^/kg)
Fracture conductivity	9 (1.22x10^-14^)	md-ft (m^2^-m)
Fracture height	65 (18.2)	ft (m)
Matrix permeability	0.00035 (6.125x10^-19^)	md (m^2^)
Matrix porosity	0.06	fraction
Horizontal wellbore length	1000 (304.8)	ft (m)

### Effect of matrix permeability

**Fig 5:**
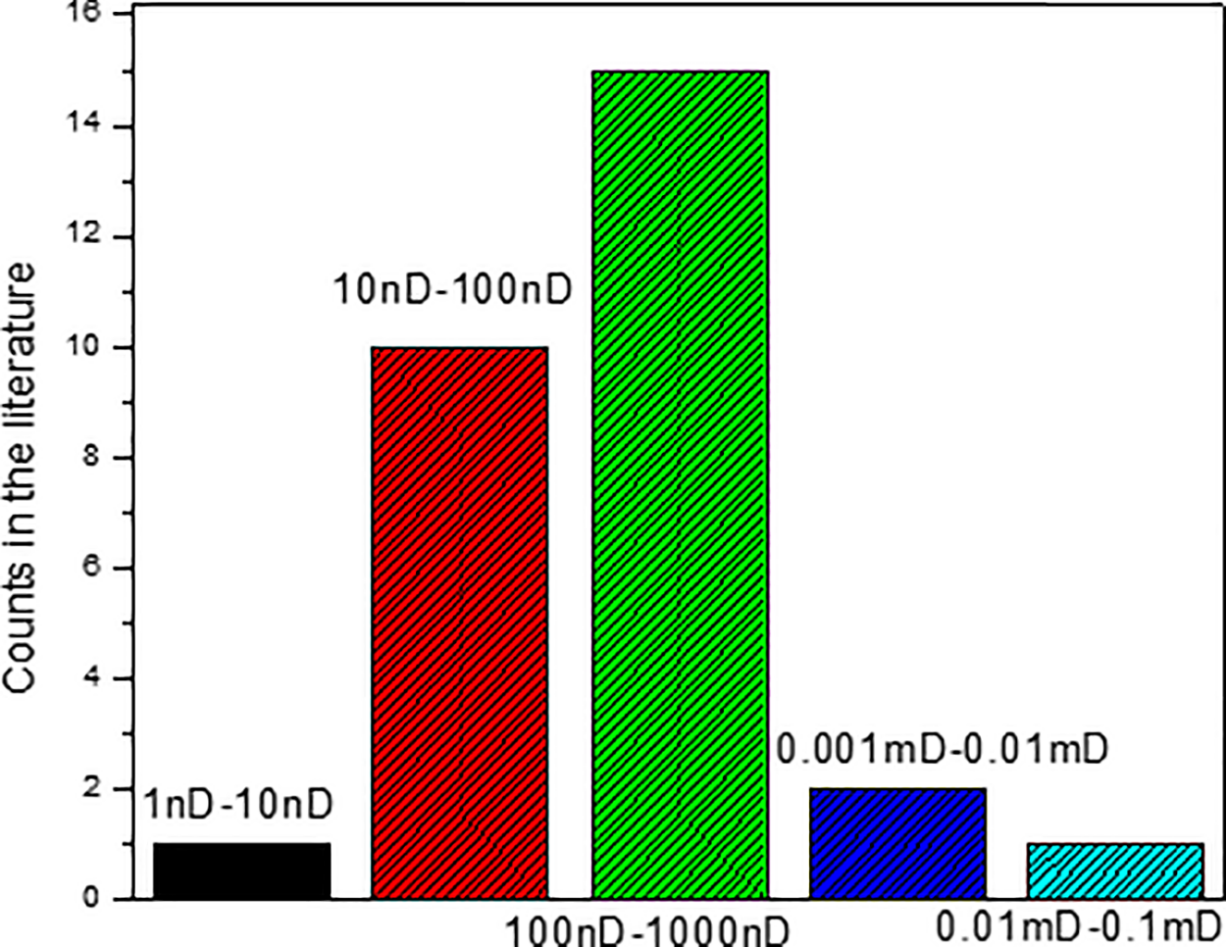
Frequency distribution diagram of shale matrix permeability.

Shale gas reservoirs are typical unconventional reservoirs with ultra-low permeability from 10 to 1000 nanodarcies (10^−6^ mD) [3]. In this sensitivity analysis, the values of this parameter was adjusted based on its actual observed range. According to the literature review, it can be found that matrix permeability of SGRs in the U.S. are in the range of 10 nD (1 nD = 10^−9^ Darcy = 10^−6^ mD) to 1000 nD. The permeability distribution is shown in Fig 5. Based on the reviewed range of matrix permeability, sensitivity analysis of matrix permeability on production performance of SGRs has been conducted. In this study, we have considered five cases with matrix permeability close to the reviewed matrix permeability range. The matrix permeability of the five cases are: 10 nD, 200 nD, 500 nD, 800 nD, and 1000 nD.

**Fig 6:**
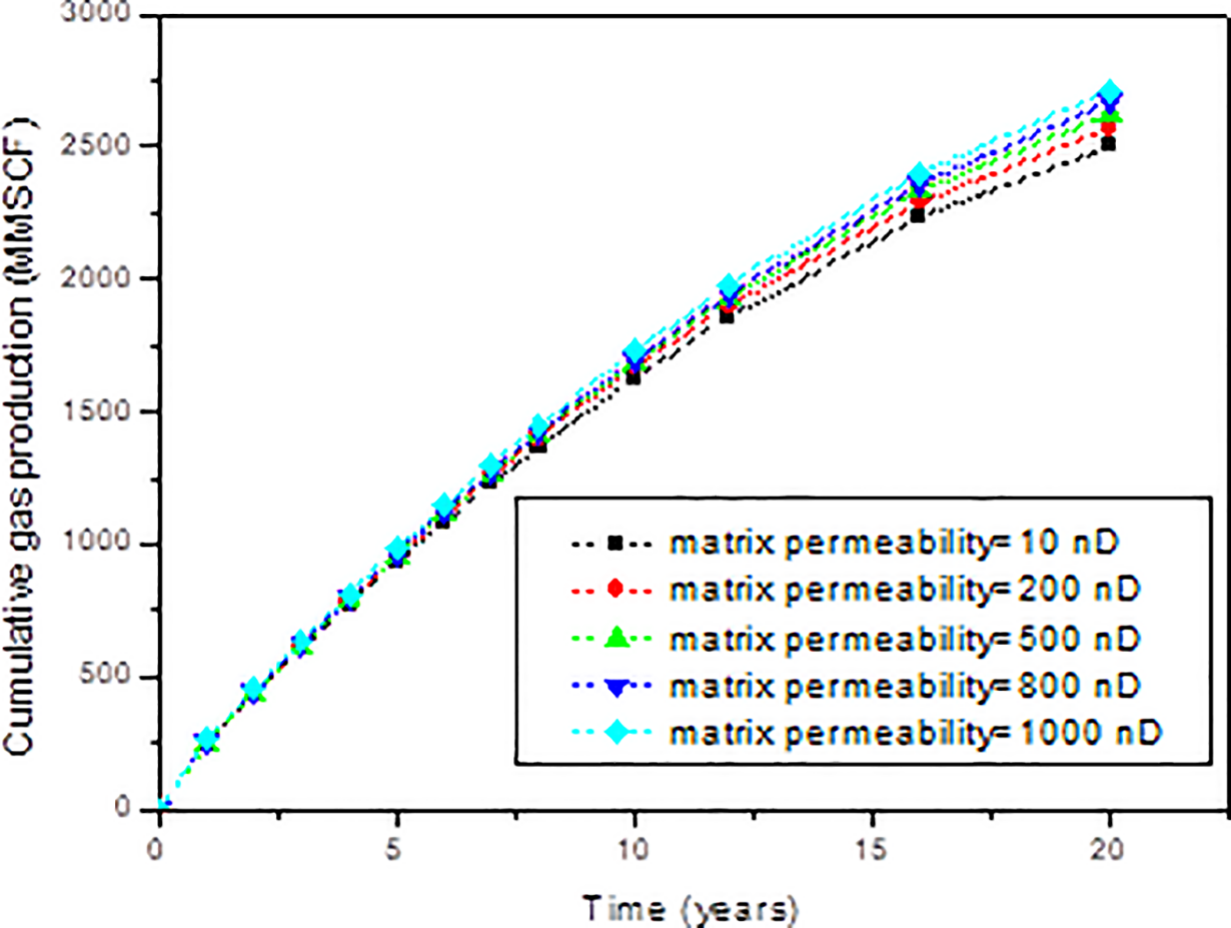
Effect of matrix permeability on the cumulative gas production of shale gas reservoirs.

The effect of matrix permeability on cumulative gas production is shown in Fig 6. The effect of matrix permeability on gas production rate can be obtained from the slope of Fig 6. From this figure, it can be found that the effect of matrix permeability on shale gas production performance is not significant. Also, it can be found that in the early period, the matrix permeability barely has influence on gas production. In the late period of gas production, the difference began to appear. However, the difference is not significant. When the matrix permeability changes from 10 nD to 1000 nD, the cumulative gas production in 20 years only increases from 2501 MMSCF to 2714 MMSCF, which is about 7.9% increase.

### Effect of matrix porosity

**Fig 7:**
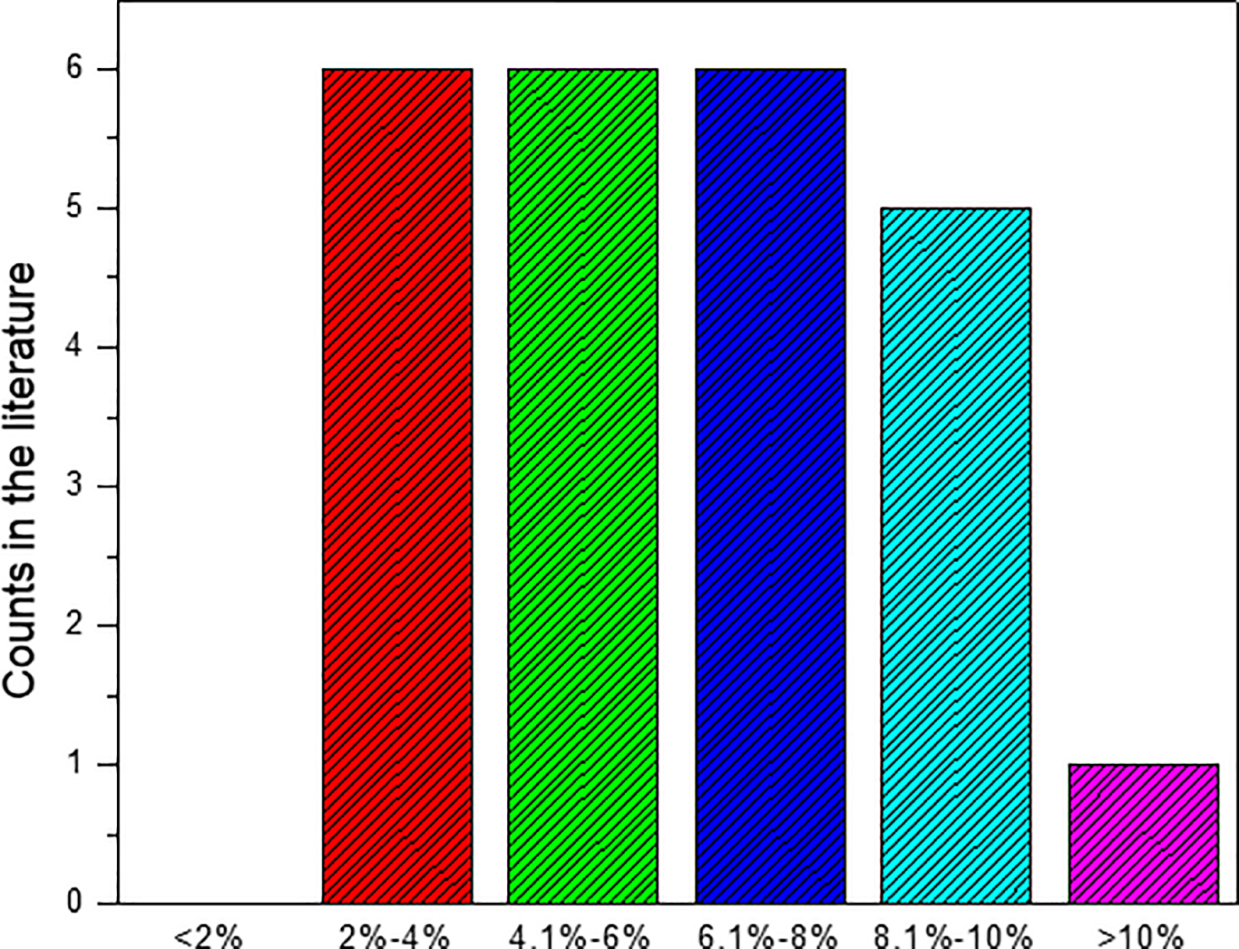
Frequency distribution diagram of shale matrix porosity.

**Fig 8:**
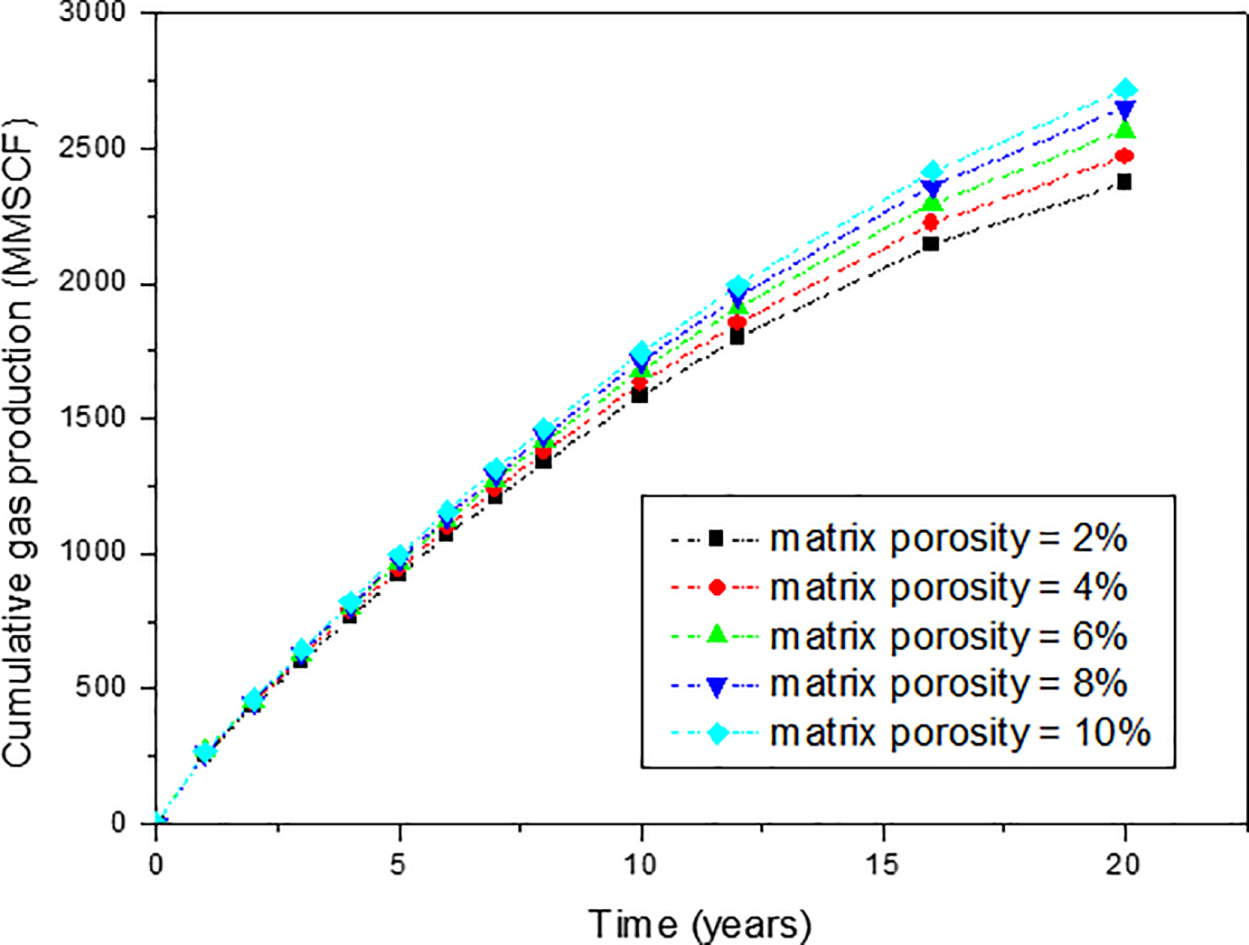
Effect of matrix porosity on the cumulative gas production of shale gas reservoirs.

Sensitivity analysis of matrix porosity on production performance of SGRs has also been conducted based on the reviewed range of matrix porosity, which is shown in Fig 7. It can be found that matrix porosity are in the range of 2% to 8%. So, in this study, five cases with matrix porosity close to this range have been considered. The matrix porosity of the five cases are: 2%, 4%, 6%, 8%, and 10%. The effect of matrix porosity on cumulative gas production is shown in Fig 8. The effect of matrix porosity on gas production rate can be obtained from the slope of Fig 8.

From Fig 8, it can be found that the effect of matrix porosity on the production performance of SGRs is also not very significant. When the matrix porosity changes from 2% to 10%, there is no significant change in the early and late production period. The cumulative gas production changes from 2372 MMSCF to 2718 MMSCF, which is 12.7% increase.

### Effect of fracture spacing

**Fig 9:**
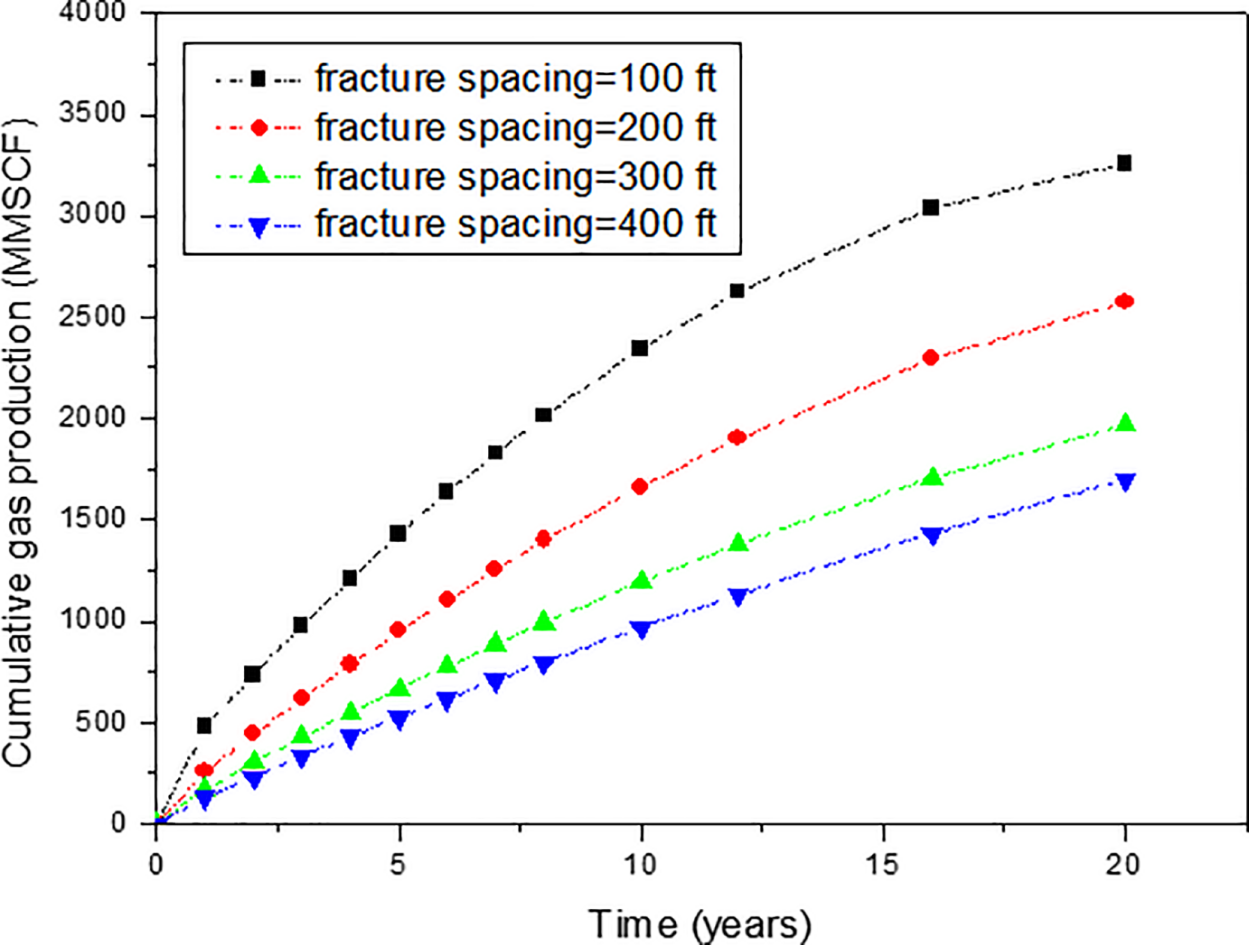
Effect of fracture spacing on the cumulative gas production of shale gas reservoirs.

When there are multiple transverse fractures in SGRs, it is important to consider the effect of fracture spacing. Cipolla et al. (2009) found that in order to achieve the commercial production rates and optimum depletion of shale gas reservoir, the fracture spacing should be minimized [27]. However, when the fracture spacing is too small, the interaction between fractures will become more apparent, which is not good for production. Also, more fractures will require more investment to create fractures. So, it is important to study the effect of fracture spacing on shale gas production performance. Sensitivity analysis of fracture spacing on production performance of SGRs has been conducted in this paper. Due to limitations of the reservoir size, we only studied four cases with fracture spacing equal to 100 ft, 200 ft, 300 ft, and 400ft. The effect of fracture spacing on cumulative gas production is shown in Fig 9. The effect of fracture spacing on gas production rate can be obtained from the slope of Fig 9.

From Fig 9, it can be found that the effect of fracture spacing on gas production performance is very significant. Difference exist not only in the early period, but also in the late period. And when the fracture spacing changes from 100 ft to 400 ft, the cumulative gas production increases from 1699 MMSCF to 3259 MMSCF, which is about 47.9% increase. Also, from the results, we can find that the smaller the fracture spacing, the more cumulative gas production is. These findings coincide with the results of Cipolla et al. (2009) [27].

### Effect of fracture half-length

**Fig 10:**
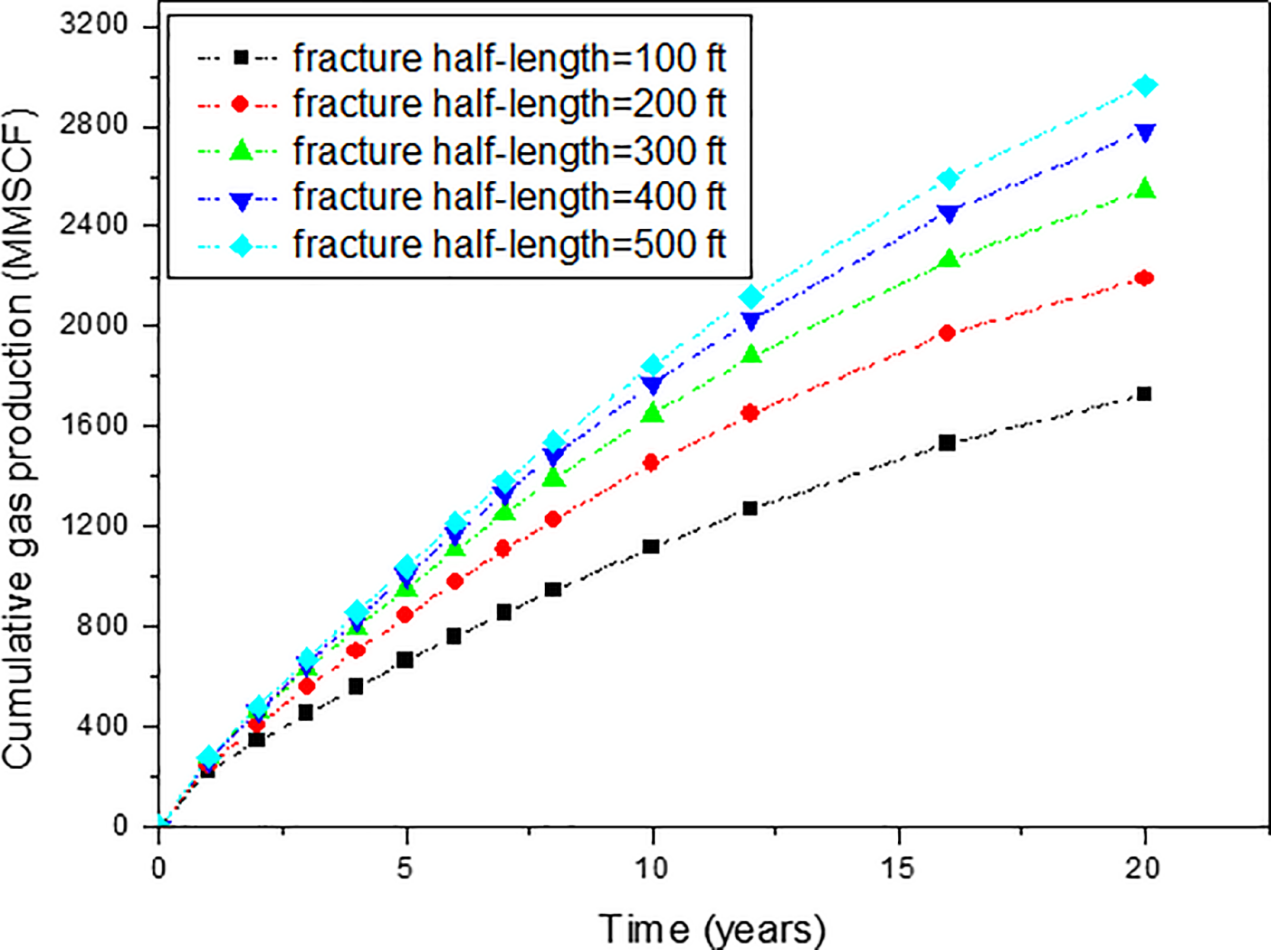
Effect of fracture half-length on the cumulative gas production of shale gas reservoirs.

Fracture half-length is the horizontal distance from horizontal wellbore to the end of hydraulic fracture. Fracture half-length is important in deciding production from multi-stage hydraulic fracturing horizontal well as this is the main high conductivity channels for fluid flow from the reservoir into the horizontal wellbore. Sensitivity analysis of fracture half-length on production performance of SGRs has been conducted in this study. We have considered five possible cases with considering limitation of the reservoir size. The fracture half-length of the five cases are: 100 ft, 200 ft, 300 ft, 400 ft, and 500 ft. The effect of fracture half-length on cumulative gas production is shown in Fig 10. The effect of fracture half-length on gas production rate can be obtained from the slope of Fig 10.

From Fig 10, it can be found that the larger the fracture half-length, the higher the cumulative gas production is. This is because the high-permeability hydraulic fractures are the main flow channels for fluid flow. Increasing the fracture half-length will increase the stimulated reservoir volume, which will contribute the gas production rate and cumulative production. As shown in Fig 10, when the fracture half-length changes from 100 ft to 500 ft, the cumulative gas production changes from 1728 MMSCF to 2965 MMSCF for the 20 years gas production, which is about 41.7% increase.

From the comparison of above four parameters, it can be found that hydraulic fracture parameters are the dominate factors in shale gas production with multi-stage hydraulic fracturing horizontal well. Matrix porosity is more dominant compared with matrix permeability, which denotes that shale matrix is mainly the storage media instead of the fluid flow media. And fracture spacing is more dominate compared with fracture half-length. In order to optimize gas production from SGRs, it is better to decrease the fracture spacing. However, it should also be noted that when the fracture spacing is decreased, more investment will be needed to create the fractures. So, the optimal plan will be the balance between technology and the economic investment.

## Conclusions

In this work, we made an attempt to study the influence of various parameters on the production performance of ultra-low permeability SGRs with multi-stage hydraulic fracturing horizontal well.

- A dual porosity dual permeability shale gas reservoir simulation model was constructed to simulate gas production from shale reservoirs with multi-stage hydraulic fracturing horizontal well with considering multiple flow mechanisms, including: viscous flow, slip flow, Knudsen diffusion, and gas desorption.
- The mathematical model was verified against the field data from the Barnett shale using the original data obtained through a previous history match. A synthetic model has been constructed to analyze the effect of reservoir parameters and hydraulic fracture parameters on gas production performance of SGRs.
- According to the sensitivity analysis, it can be found that hydraulic fracture parameters are the dominant factors which affect shale gas production. Fracture spacing is more dominant compared with the fracture half-length, while matrix porosity is more dominant compared with matrix permeability.

## Nomenclature

*▽p* = pressure gradient, Pa/m

p_f_ = fracture pressure, Pa

*p*_*m*_ = matrix pressure, Pa

*K* = permeability, m^2^

*k*_*mi*_ = intrinsic matrix permeability, m^2^

*k*_*fi*_ = initial fracture permeability, m^2^

J_v_ = mass flow rate, kg/(m^2^⋅s)

J_k_ = mass flow caused by Knudsen diffusion, kg/(m^2^·s)

C_m_ = gas mole concentration, mol/ m^3^

*ν* = velocity, m/s

σ = crossflow coefficient between the matrix and fracture systems

*V*_*L*_ = Langmuir volume, m^3^/kg

*P*_*L*_ = Langmuir pressure, Pa

μ_g_ = gas viscosity, Pa∙s

*r*_*w*_ = wellbore radius, m

*r*_*e*_ = effective radius, m

*q* = mass production rate, m^3^/s

*q*_*v*_ = volume production rate, kg/s

∅_f_ = fracture porosity, %

∅_*m*_ = matrix porosity, %

*p*_*wf*_ = bottom hole pressure, Pa

$\bar{p_{f}}$ = average pressure in the fracture system, Pa

Γ_1_ = Outer boundary domain

Γ_2_ = Inner boundary domain

*t* = time, s

*x*, *y*, *z* = cartesian coordinate

L_x_, L_y_, L_z_ = fracture spacing in the *x*, *y*, *z* directions

ρ_s_ = density of shale core, kg/m^3^

q_a_ = adsorption gas per unit area of shale surface, kg/m^3^

q_std_ = adsorption gas volume per unit mass of shale, m^3^/kg

V_std_ = gas mole volume under standard condition, m^3^/mol

*α* = tangential momentum accommodation coefficient

## Supporting information

S1 File The data set for Figs 3 and 5–10 are included. (ZIP)
